# Enhancing the Detection of BOLD Signal in fMRI by Reducing the Partial Volume Effect

**DOI:** 10.1155/2014/973972

**Published:** 2014-03-09

**Authors:** Yiping P. Du, Renxin Chu, Jason R. Tregellas

**Affiliations:** ^1^Department of Biomedical Engineering, Zhejiang University, Hangzhou 310027, China; ^2^Key Laboratory for Biomedical Engineering of Education Ministry of China, China; ^3^Department of Psychiatry, Brain Imaging Center, University of Colorado School of Medicine, Aurora, CO, USA; ^4^Research Service, Denver VA Medical Center, Denver, CO 80220, USA

## Abstract

*Purpose*. To investigate the advantages of reducing the partial volume effect (PVE) to enhance the detection of the BOLD signal in fMRI. *Methods*. A linear phase term was added in *k*-space to obtain half-voxel shifting of 64 × 64 T_2_*-weighted echo-planar images. Three sets of image data shifted in the *x*, *y*, and diagonal direction, respectively, are combined with the original 64 × 64 data to form the 128 × 128 voxel-shifted interpolated data. *Results*. A simulation of a synthetic fMRI dataset shows that the voxel-shifted interpolation (VSI) can increase the *t*-score up to 50% in single-voxel activations. An fMRI study (*n* = 7) demonstrates that 20.4% of the interpolated voxels have higher *t*-scores than their nearest neighboring voxels in the original maps. The average increase of the *t*-score in these interpolated voxels is 13.3%. *Conclusion*. VSI yields increased sensitivity in detecting voxel-size BOLD activations, improved spatial accuracy of activated regions, and improved detection of the peak BOLD signal of an activated region. VSI can potentially be used as an alternative to the high-resolution fMRI studies in which reduction in SNR and increase in imaging time become prohibitive.

## 1. Introduction

Functional MRI (fMRI) studies with the blood oxygenation level-dependent (BOLD) contrast [1, 2] typically use a spatial resolution of a few millimeters (e.g., with an in-plane resolution of 2–4 mm and through-plane resolution of 3–6 mm). It is desirable to increase the spatial resolution in fMRI for at least two reasons. First, the spatial localization of neuronal activation can be improved with a higher spatial resolution [3, 4]. The gray matter (GM) typically has a thickness of 2-3 mm in human brains. Even within a cortical layer of GM, the distribution of venous vessels is heterogeneous and, therefore, the BOLD contrast can be increased by using a voxel size as small as 1 mm^3^ [5–7]. Secondly, the BOLD response originating from sub-voxel activation can be increased by reducing the voxel size. For example, it was demonstrated that BOLD activation in the hippocampal formation is often undetectable at a resolution of 4 × 4 × 5 mm^3^ but can be reliably detected at a higher resolution of 2 × 2 × 3 mm^3^ [8]. In fMRI studies designed to examine midbrain nuclei [9], single-voxel BOLD activation is not unusual because of the small size of these nuclei.

**Figure 1:**
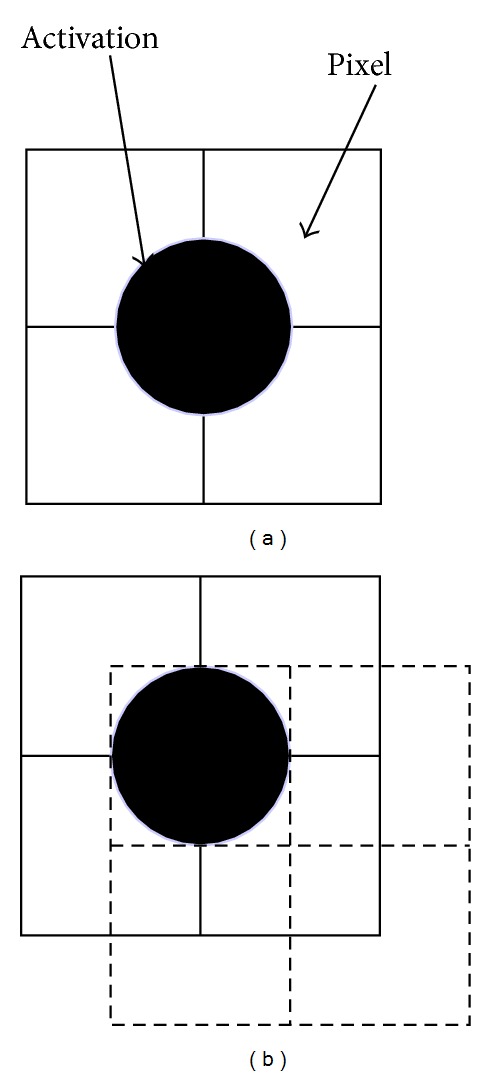
A scheme illustrates that the detection of BOLD signal is greatly affected by the location of the activated region relative to the voxels sampled when the region of activation, represented as a filled circle, is comparable to the size of a voxel, represented by the squares. When the distance of an activated region from its nearest voxel is close to half of the voxel size, the measured BOLD signal will be substantially reduced (a). Diagonally shifting the location of voxels by half of the voxel size, as shown in the dashed squares in (b), can greatly reduce the PVE and improve the detection of BOLD signal.

The ability to detect small region (e.g., single-voxel size) BOLD activation is highly dependent on the location of the activation. The detected BOLD response is highest when the activation site is located at the center of a voxel and is lowest when the activation is located at the corners of 4 neighboring voxels in two-dimensional data (see Figure 1(a)) or 8 neighboring voxels in three-dimensional data. Similarly, the detected BOLD response at the edge of an activated region is dependent on the relative location of the edge to the voxels. This dependency of detected BOLD response on the relative location of the BOLD activation to the grid of voxels is determined by the voxel sensitivity function [10] and is referred to as the partial volume effect (PVE). PVE occurs in MRI when a voxel is only partially filled with the source of imaging contrast.

PVE can be reduced by acquiring images with high spatial resolution. High spatial resolution is commonly achieved by increasing the acquisition matrix of fMRI data, because any reduction of field-of-view (FOV) is limited by the dimension of the imaging objects or region of interest. Several studies have demonstrated the advantages of higher spatial resolution fMRI, including the increased spatial specificity of BOLD activations [4–6] and suppression of the BOLD signal originating from large vessels [3]. The acquisition of high-resolution fMRI data, however, inevitably reduces spatial SNR and prolongs acquisition time. With the current echo-planar imaging (EPI) or spiral imaging technology, a low resolution (e.g., with a 64 × 64 matrix) whole-brain dataset can be acquired with a repetition time (TR) on the order of 2 seconds [11]. Acquiring whole-brain fMRI with a higher spatial resolution (e.g., with a matrix size larger than 128 × 128) will considerably reduce the temporal resolution and spatial SNR, resulting in a greatly reduced statistical power in fMRI data analysis. This reduced statistical power can make the high-resolution approach unfavorable or even prohibitive to most fMRI studies. Furthermore, event-related fMRI studies require a TR of 2 seconds or shorter in order to adequately sample the hemodynamic response, making high-resolution acquisition even less useful for these studies. Additionally, image distortion induced by field inhomogeneity becomes more problematic in single-shot high-resolution echo-planar images [12].

In fMRI, PVE can be the result of three conditions: (1) when the size of the activated region is smaller than the voxel size, (2) when the peak of the true BOLD activation is mismatched from the location of any voxel, and (3) when the voxels at the boundary of activated regions are only partially occupied by the activation. When the dimension of an activation site is similar to the size of a voxel and the location of the activation is not centered on any one voxel (Figure 1(a)), the second and third conditions above can become the primary sources of PVE. In this case, the averaging of the BOLD signal with nonactivated partial volume greatly dilutes the BOLD contrast. When the BOLD contrast is diluted below certain threshold, the activated region becomes undetectable.

In this study, we hypothesize that spatial resampling with a half-voxel shift in the image space can greatly reduce PVE (Figure 1(b)) and enhance the ability to detect the activation through a better spatial match. In this paper, both simulation and fMRI experiments demonstrate that reducing PVE with voxel-shifted interpolation can substantially improve the detection of BOLD signal in fMRI.

## 2. Materials and Methods

### 2.1. Voxel-Shifted Interpolation

**Figure 2:**
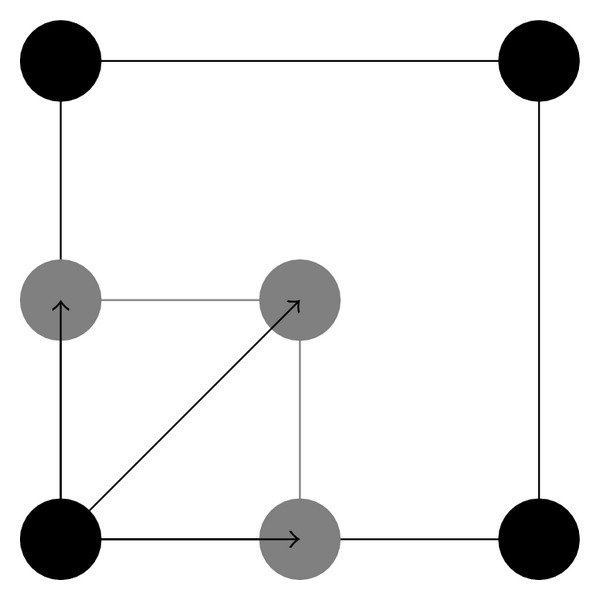
The scheme illustrates the voxel-shifting approach. The dark dots represent the centers of four original voxels. The gray dots represent the centers of three interpolated voxels shifted from the original voxel at the lower-left corner.

In the voxel-shift interpolation (VSI), three image sets with a 64 × 64 matrix were reconstructed with a half-voxel shift along the *x*, *y*, and diagonal direction, respectively, in addition to the original 64 × 64 image. According to the Fourier shift theorem, these shifts were implemented by applying a linear phase term in the complex *k*-space data in the corresponding directions:

(1) $$\begin{array}{c} \Phi \left(\kappa \right)=\frac{2\pi \kappa }{N}, \end{array}$$

where half-integer *κ* = −*N*/2 + 1/2,…, *N*/2 − 1/2 and *N* is the matrix size of the original image. Finally, the voxels in the three shifted image matrices were interspersed with those in the original image, based on the relative shift that was applied to each of the shifted image, to form an interpolated image with a 128 × 128 matrix size (see Figure 2).

VSI can also be implemented by filling zeroes in the* k*-space and is thus also referred to as zero-filled interpolation [13] or by sinc interpolation with complex images. Although the names of VSI, zero-filled interpolation, and sinc interpolation suggest different approaches of implementation, they are nevertheless mathematically equivalent. We use the term VSI in this paper, as it provides a pictorial description of the interpolation that can better illustrate the reduction of PVE.

### 2.2. Simulation

**Figure 3:**
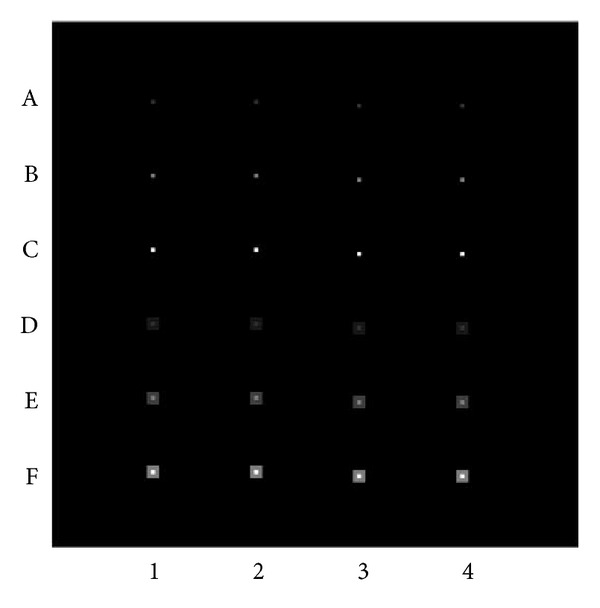
A synthetic data set (128 × 128) simulating fMRI activations of various sizes. Single-voxel “activations,” with signal changes of 1%, 2.5%, and 5% were placed at rows A, B, and C, respectively. The 2D coordinates of activation at column 1 are odd, odd; column 2 are even, odd; column 3 are odd, even; and column 4 are even, even, respectively. Rows D to F show multivoxel “activations”: the same single-voxel “activations” in rows A to C were duplicated, together with 8 surrounding “activated” voxels added to these single-voxel “activations.” The signal of the surrounding voxels is half of that in the central voxel.

A synthetic dataset mimicking fMRI activation was generated for simulation. The data set had a matrix size of 128 × 128, with 240 time points, alternating “ON” states for 30 time points and “OFF” states for 30 time points. The background intensity was set at 10,000 in the real component and zero in the imaginary component. The data set had an SNR of 500 with Gaussian noise introduced in both real and imaginary components. The SNR of the synthetic data was intentionally set higher than that typically used in fMRI scans in order to better demonstrate the improvement in detecting “activation” through reducing PVE. In the “ON” state, a single-voxel “activation,” with a signal of 1%, 2.5%, and 5%, respectively, was placed at 4 locations. The 2D coordinates of these 4 locations are in the combination of odd, odd; even, odd; odd, even; and even, even, respectively, as shown in the upper part of Figure 3. The same “activated” voxels were duplicated at the lower part of the figure, with surrounding “activated” voxels being added to these single-voxel “activations.” The “activation” signal of each of the surrounding voxels was half of that in the central voxel. The simulation of the single-voxel “activation” was to mimic the activations in mid-brain nuclei, while the simulation of the multivoxel “activation” was to demonstrate the effect of PVE in cortical activations.

A two-dimensional Fourier transform was applied to the dataset to generate the *k*-space data. The central portion of the *k*-space data with a size of 64 × 64 was used to reconstruct the 64 × 64* original *images. The same data set was also used to reconstruct the* shifted* images using VSI to form the 128 × 128* interpolated* images. *T*-scores of each voxel in the original and the interpolated images were calculated using the general linear model in SPM2b software package (Wellcome Department of Cognitive Neurology, University College London, UK). A *t*-score of 3.18 was used to threshold the “activation” maps.

### 2.3. fMRI Experiments

Functional MRI data were acquired using T_2_*-weighted gradient-echo EPI on a 3T MRI scanner (General Electric, Waukesha, WI), while subjects (*n* = 7, male/female = 3/4, age = 36.3 ± 10.5 years old) were performing a smooth pursuit eye movement (SPEM) task [14–16], under a protocol approved by a local institutional review board. The imaging parameters were TE/TR/flip angle = 32 ms/2500 ms/77°, 24 slices, field-of-view = 22 cm, slice thickness = 4 mm, and matrix size = 64 × 64. The task was run twice; each consisted of task (30 s)/rest (30 s) for 4 cycles.

Functional images were reconstructed offline from the *k*-space data using a software package developed by our lab. The original image set with a 64 × 64 matrix was reconstructed directly from the acquired data. VSI was applied to the *k*-space data to obtain the 128 × 128 interpolated images.

VSI was also applied to the original* magnitude* images for comparison. The magnitude VSI is an approximation of the *k*-space VSI because the magnitude fMRI data are more accessible in scanners. The magnitude VSI can also be used to retrospectively interpolate the fMRI data acquired without saving the complex *k*-space data. In this approach, an original 64 × 64 magnitude image was used as the real component of the complex image and the imaginary component of the complex image was set to zero. Fourier transform was applied to the complex images to generate the 64 × 64 *k*-space data prior to VSI to a 128 × 128 matrix.

### 2.4. fMRI Data Analysis

fMRI data analysis was performed using the SPM2b software package. A *t*-score was calculated for each individual voxel. The same procedure was used in the data analysis to ensure the comparability of results between the original images with a 64 × 64 matrix and the interpolated images. The procedure is not involved in any spatial interpolation or smoothing. In order to focus on the comparison between the interpolated and original fMRI data and avoid unnecessary variables in the comparison, no other preprocessing procedures, such as motion correction or normalization to standard space, were performed prior to the fMRI data analysis. These steps involve additional spatial interpolation and can further complicate the comparisons of interest in this study. The data from one of the subjects was discarded due to excessive motion during data acquisition. A *t*-score of 3.18 (*P* ≤ 0.000022) was used as the threshold of activation and the same scale of *t*-score (i.e., from 3.18 to 22.83) was used in the display of the statistical parametric mapping results.

## 3. Results

**Figure 4:**
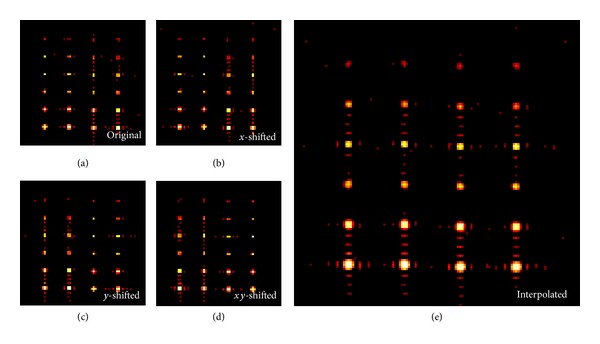
Simulation results of VSI represented as *t*-score maps of the “activations” using the template shown in Figure 3. (a) is the original 64 × 64 map (without VSI) obtained from the central portion (64 × 64) of the *k*-space of the synthetic data set shown in Figure 3, (b) is the 64 × 64 map shifted half-voxel along the horizontal direction, (c) is the 64 × 64 map shifted half-voxel along the vertical direction, and (d) is the 64 × 64 map shifted half-voxel along the diagonal direction. It is observed that the left column is blurred horizontally in (b), vertically in (c), and both horizontally and vertically in (d). The left column has the least blurring and typically has the highest *t*-scores in (a). The same column has the most severe burring and typically has the lowest *t*-scores in (d). The voxel-shifted interpolated 128 × 128 map (e) is the interspersed combination of (a), (b), (c), and (d). The location-dependent blurring and reduction of *t*-score seen in (a)–(d) was largely eliminated in (e). The SNR of the images is approximately 500 with Gaussian noise introduced to the complex *k*-space.

Simulation results show that the activated regions have different degrees of blurring in the* original* 64 × 64 images (see Figure 4(a)), depending on whether their *x*- and *y*-coordinates are even or odd numbers. The activations located at odd, odd coordinates (left column) have the least blurring and more likely have the highest *t*-scores. The activations located at even, even coordinates (right column) have the most severe blurring and more likely have the lowest *t*-scores. The activations located at even, odd coordinates are blurred horizontally and the activations located at odd, even coordinates are blurred vertically (see the middle two columns). These activations likely have intermediate *t*-scores. In the 64 × 64 images shifted along *x*-, *y*-, and *xy*-directions, similar spatial dependency of the *t*-scores on the relative location of the activations to the voxels is observed, except that the peak *t*-scores are at the second column from the left in the *x*-shifted image (Figure 4(b)), the third column from the left in the* y*-shifted image (Figure 4(c)), and the right column in the *xy*-shifted image (Figure 4(d)), respectively. In the interpolated 128 × 128 image (Figure 4(e)), this spatial dependency of *t*-scores is largely removed. The residual variation of the *t*-scores in different columns is caused by the added noise. As expected, all the activations were spatially blurred due to the 64 × 64 truncation of the *k*-space data prior to the interpolation.

**Figure 5:**
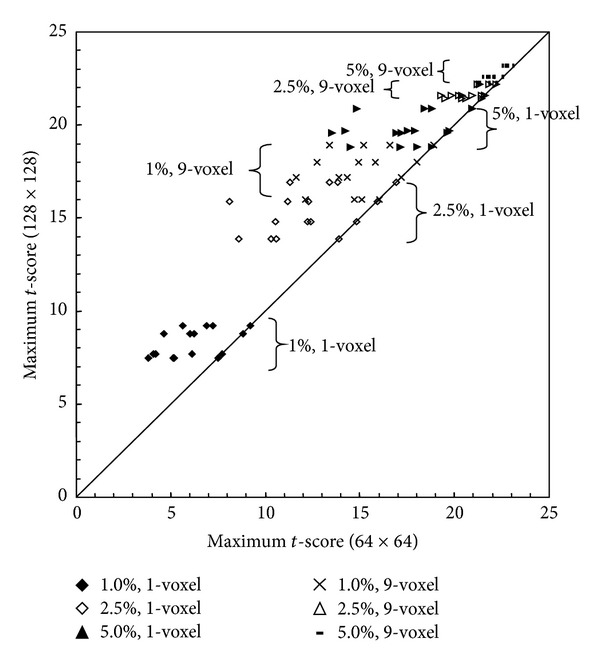
The measurement of peak *t*-score in each of the activations in the simulated data is shown in this plot. For each of the 24 activated regions, the *y*-axis represents the maximum *t*-score in the interpolated 128 × 128 map. The *x*-axis represents the maximum *t*-score in each of the four 64 × 64 maps. The highest of the four *t*-scores in each region is the same as the maximum *t*-score in that region in the interpolated map. This plot shows that the VSI can increase the *t*-score by up to 50% in single-voxel activations with a signal change of 1% (solid diamonds). The increase of *t*-score with VSI is substantial for single-voxel activations with a signal of up to 5% and 9-voxel activations with a signal change of 1%.

Figure 5 is the scatter plot demonstrating the increase of peak *t*-scores in the interpolated 128 × 128 images (*y*-axis) compared to the peak *t*-score in the original and shifted 64 × 64 images (*x*-axis) for each of the 24 activations as in Figure 3. Up to a 50% increase in the maximum* t*-score of the 1% single-voxel activation was observed (see the lower-left corner of the plot). Substantial increase of the peak *t*-score can also occur with VSI in the single-voxel activations with higher BOLD signal and multivoxel activation with lower BOLD signal, as shown in the middle part of the plot. This increase of peak *t*-score with VSI becomes modest in multivoxel activations with high BOLD signal, as shown in the upper-right corner of the plot. These simulation results indicate that VSI can substantially improve the detection of small activated regions with a low BOLD signal. This feature is quite relevant in real fMRI studies, because small activated regions usually have a lower BOLD signal and are more likely undetectable in fMRI analysis.

**Figure 6:**
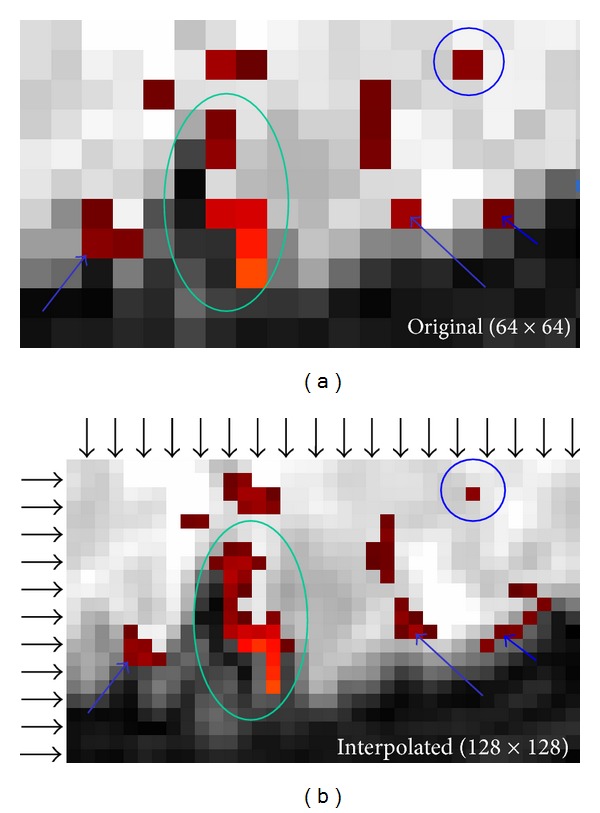
An example of the original (a) and *k*-space interpolated (b) activation maps at the visual cortex. The rows and columns indicated by the black arrows in the interpolated map consist of the voxels obtained through voxel-shifting. The interpolated map would be reduced to the original map if these arrow-indicated rows and columns were removed. In this example, a single voxel activation in the original map could be a single voxel (circle), a group of 6 voxels (long arrow), or a strip of 7 voxels (short arrow) in the interpolated map. Two apparently separated activated areas in the original map are connected in the interpolated map (ellipse). Two 6-voxel activated regions with similar shape in the interpolated map are represented as a single-voxel (long arrow) activation or a 3-voxel activation (medium arrow) in the original map.

Human fMRI experiments also show substantial improvement using VSI to detect BOLD signal response. An example of the original (a) and interpolated (b) activation map at the visual cortex is shown in Figure 6. In this example, a single voxel activation in the original map can be a single-voxel (circle), a group of 6 voxels (short arrow), or a strip of 7 voxels (long arrow) in the interpolated map. Two apparently separated activated areas in the original map are connected in the interpolated map (ellipse). On the other hand, two similar 6-voxel activated regions in the interpolated map (long and medium arrows) appear as single-voxel and 3-voxel activated regions in the original map. In Figure 6(b), the voxels in the rows and columns indicated by the black arrows are obtained through VSI. The other voxels, one out of four in any 2 × 2 cluster, are from the original image.

**Figure 7:**
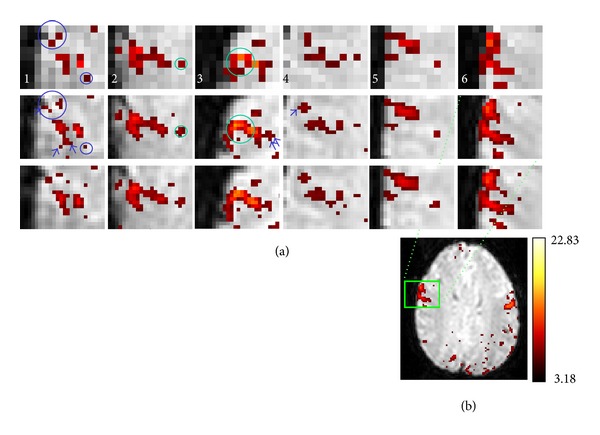
The activation maps at the frontal eye fields (FEF) in 6 subjects. In (a), the original maps are shown at the top, the interpolated maps (*k*-space) are shown at the middle, and the interpolated maps (magnitude) are shown at the bottom. Two apparently connected activated voxels in the original map are separated in the interpolated map (large blue circle). A single-voxel activation in the original maps can be a single-voxel activation (small blue circle) or a group of 6 activated voxels (small green circle) in subjects 1 and 2 in the interpolated map. In subject 3, the activated voxels form an enclosed loop in the original map but not in the interpolated map (large green circle). Similar results were also seen in the interpolated maps obtained with magnitude VSI. (b) shows the interpolated map in a slice from subject 6, at which the region of FEF is indicated by a green box.

The activation maps at the frontal eye fields (FEF) in 6 of the 7 subjects are shown in Figure 7. In this figure, the original maps are at the top, the maps interpolated with *k*-space VSI are in the second row, and the maps interpolated with magnitude VSI are at the third row. In subject 1, two apparently connected activated voxels in the original map are separated in the interpolated map (large blue circle). A single voxel activation in the original maps can be a single voxel activation (small blue circle in subject 1) or a group of 6 activated voxels (small green circle in subject 2) in the interpolated maps. In subject 3, the activated voxels apparently form an enclosed loop in the original map (large green circle) but not in the interpolated map. In general, the activation with VSI confirms better to the known gyral anatomical location of the FEF acquired with a high-resolution SPEM study [17].

**Figure 8:**
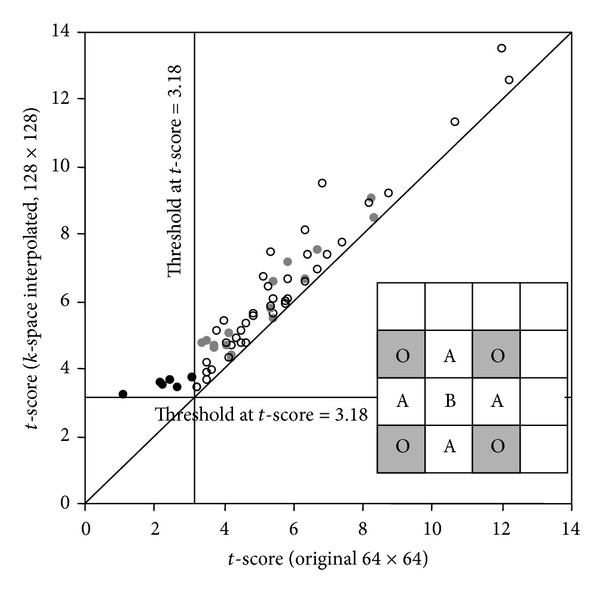
The *t*-scores are evaluated in the original and *k-*space interpolated activation maps in the FEF region in six subjects. The inset drawing shows the spatial relationship of the A- and B-type voxels in the interpolated image to the location of the original voxels, labeled as Os. Each circle in this plot shows an interpolated voxel that has a higher *t*-score (*y*-axis) than the highest *t*-score of its nearest original neighbors (*x*-axis), either an A-type (open circle) or B-type (gray solid circle) voxel. In the 6 interpolated maps, the *t*-scores in 62 of a total 304 voxels are higher than the highest *t*-scores in the nearest neighboring original voxels, with an average of 13.3% increase of the *t*-score. In each of the 6 interpolated voxels shown as dark solid circles, the highest *t*-score of its nearest original neighbors is below the threshold of 3.18 in the original maps. These 6 interpolated voxels, indicated by the blue arrows in Figure 7(a), have *t*-scores above the threshold and are located at the edge of the multivoxel activated regions. The diagonal line represents the situation when the *t*-scores in the original maps are identical to that in the interpolated maps.

**Figure 9:**
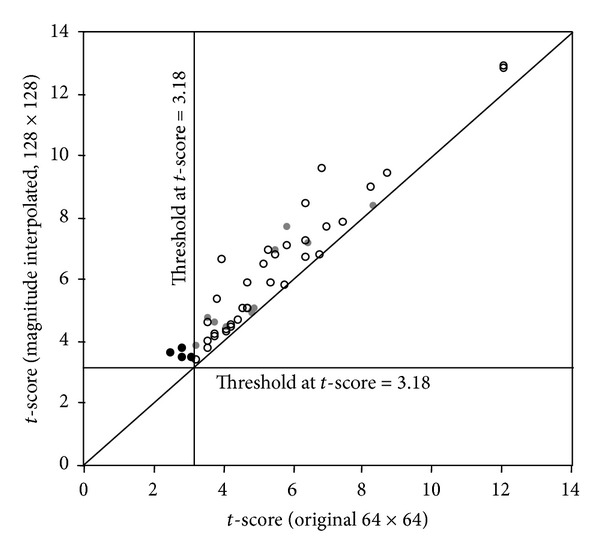
The *t*-scores are evaluated in the original and magnitude interpolated activation maps in the FEF region in six subjects. Each circle in this plot shows an interpolated voxel that has a higher *t*-score (*y*-axis) than the highest *t*-score of its nearest original neighbors (*x*-axis), either an A-type (open circle) or B-type (gray solid circle) voxel. In the 6 interpolated maps, the *t*-scores in 49 of a total 311 voxels are higher than the highest *t*-scores in the nearest neighboring original voxels, with an average of 14.4% increase of the *t*-score. In each of the 4 interpolated voxels shown as dark solid circles, the highest *t*-score of its nearest original neighbors is below the threshold of 3.18 in the original maps.

Figure 8 shows the evaluation of the *t*-scores in the *k-space* interpolated and original activation maps in the FEF region in six subjects. There were 304 voxels that had *t*-scores above the threshold and thus considered as activated voxels. An interpolated voxel with a *t*-score higher than any of its two nearest horizontal or vertical original neighbors (labeled as Os in the inset drawing) is referred to as an A-type voxel (labeled as A), while an interpolated voxel with a higher *t*-score than any of its four nearest diagonal original neighbors (labeled as B) is referred to as a B-type voxel. Each circle in this plot shows an interpolated voxel that has a *t*-score (*y*-axis) higher than the highest* t*-score of its nearest original neighbors (*x*-axis). In the 6 interpolated maps, 20.4% of the voxels (i.e., 62 out of 304 voxels) are either A- (47) or B-type (15) voxels, with an average of 13.3% increase in *t*-score. In each of the 6 A-type interpolated voxels shown as dark solid circles, none of its nearest original neighbors has a* t*-score above the threshold of 3.18. These 6 interpolated voxels have *t*-scores above the threshold and become part of the multivoxel activated regions. Single-voxel activations in the interpolated maps are excluded in the statistics. Similar evaluation of the *t*-scores was applied to the* magnitude* interpolated activation maps (see Figure 9), with 311 activated voxels. Note that the signal in each voxel is slightly different between the* k-space* interpolated data and* magnitude* interpolated data. This evaluation demonstrates that 15.8% of the voxels (i.e., 49 out of 311 voxels) are either A- (38) or B-type (11) voxels, with an average of 14.4% increase of the *t*-scores. The location of 76% A-type (31) and B-type voxels (6) in the magnitude interpolated maps are overlapped with that in the *k*-space interpolated maps.

## 4. Discussion

### 4.1. VSI versus Other Spatial Interpolation Approaches

The shifted and original images are independently reconstructed from the same *k*-space data using the same algorithm. The only difference in the reconstruction of the shifted images is the added linear phase in *k*-space prior to the inverse Fourier transform. The shifted images can be considered to carry the same amount of information and as “true” as the original images. Therefore, the interpolated fMRI data is not expected to increase false positive detection when voxel-by-voxel fMRI analysis is performed. Also, the shifted images have the same SNR and physical voxel dimension as the original images. In comparison, other spatial interpolation methods use the intensity of the neighboring voxels to generate the point data at locations not originally sampled. As such, the intensities of these spatially interpolated voxels are different than the true intensities if they were actually acquired. It is noteworthy that *k*-space VSI does not introduce additional blurring, because it does not increase the physical voxel dimension, although the displayed interpolated images appear much less pixelated. In comparison, other interpolation methods usually introduce additional spatial blurring.

Complex *k*-space VSI offers several advantages compared to the truncated sinc interpolation in the image space. First, the sinc interpolation applied to the magnitude image is only an approximation of the complex *k*-space VSI, due to the lack of phase information presented in the image data. Secondly, the kernel size of the sinc interpolation is usually severely truncated in order to perform the interpolation with reasonable computation time. Because the equivalent kernel size of VSI is equal to the matrix size of the image, both complex *k*-space VSI and magnitude VSI are superior to severely truncated sinc interpolation.

### 4.2. PVE and Its Reduction in BOLD fMRI

VSI provides a unique alternative for the detection of highly localized activation with a weak BOLD response. A voxel-size BOLD activation usually has a weak BOLD signal. For example, a previous study of the point spread function of the BOLD activation in human V1 at 4 Tesla demonstrated that both the spatial extent and the amplitude of BOLD signal decrease when the stimuli with a constant amplitude decrease in size [18]. PVE can severely hamper the detection of a voxel-size BOLD activation, due to the small size and low BOLD signal change of the activation. Figure 4 shows that PVE can thus severely reduce the spatial accuracy in depicting the activated regions in the original map, especially at the boundaries of activated regions or when the size of the activated region is in the order of voxel size. Although PVE can be reduced by increasing the spatial resolution, the reduction of SNR in high-resolution fMRI can outweigh the benefits of reduced PVE or even become prohibitive in detecting the weak BOLD signal response in some studies.

The shape of the activated region is better depicted and the apparent location of activated region, which can be defined by the center of mass, can be more precisely determined with VSI. The increased precision in locating the activated regions can potentially improve the fMRI mapping of visual cortices [19], tonotopy [20–22], and somatotopy [23]. VSI may be particularly useful in presurgical mapping studies, where the precision of anatomical location is of paramount importance.

In the simulation, the spatial dependency of the shape and maximum *t*-score on the relative location of an activation site on the grid of voxels in the low resolution images (i.e., 64 × 64) clearly demonstrate the effect of PVE on the ability to detect BOLD responses in fMRI. In comparing the spatially dependent variation of the maximum *t*-score for each of 24 activated regions in simulation, only the second condition of PVE discussed in the introduction (i.e., the peak of the BOLD activation is mismatched from the location of any voxel) was involved. The results of this simulation show that VSI is very effective in reducing PVE arising from this condition, especially when the activation is weak and highly localized.

The increased *t*-score in the A- and B-type voxels in the human fMRI experiment is the result of reducing PVE through better matching the voxel sensitivity function with the spatial profile of the BOLD signal. In six A-type voxels shown as dark solid circles in Figure 8, the *t*-scores of their nearest original neighbors are below the threshold. These A-type voxels, as indicated by the blue arrows in Figure 7, demonstrates the reduction of PVE arising from the third condition (i.e., the voxels at the boundary of activated regions are partially occupied by the activation) and improvement in delineating the boundary of activated regions.

In the magnitude interpolated maps, the A- and B-type voxels consist of 15.8% of the total activated voxels, comparable to 20.4% in *k*-space interpolated maps. Furthermore, 76% of these A- and B-type voxels are at the same location as the A- or B-type voxels in the* k*-space interpolated maps. These comparisons suggest that the magnitude interpolation is a good approximation of the* k*-space interpolation and thus puts forward this VSI method as a viable postprocessing option for conventional fMRI image data.

### 4.3. Other Considerations

**Figure 10:**
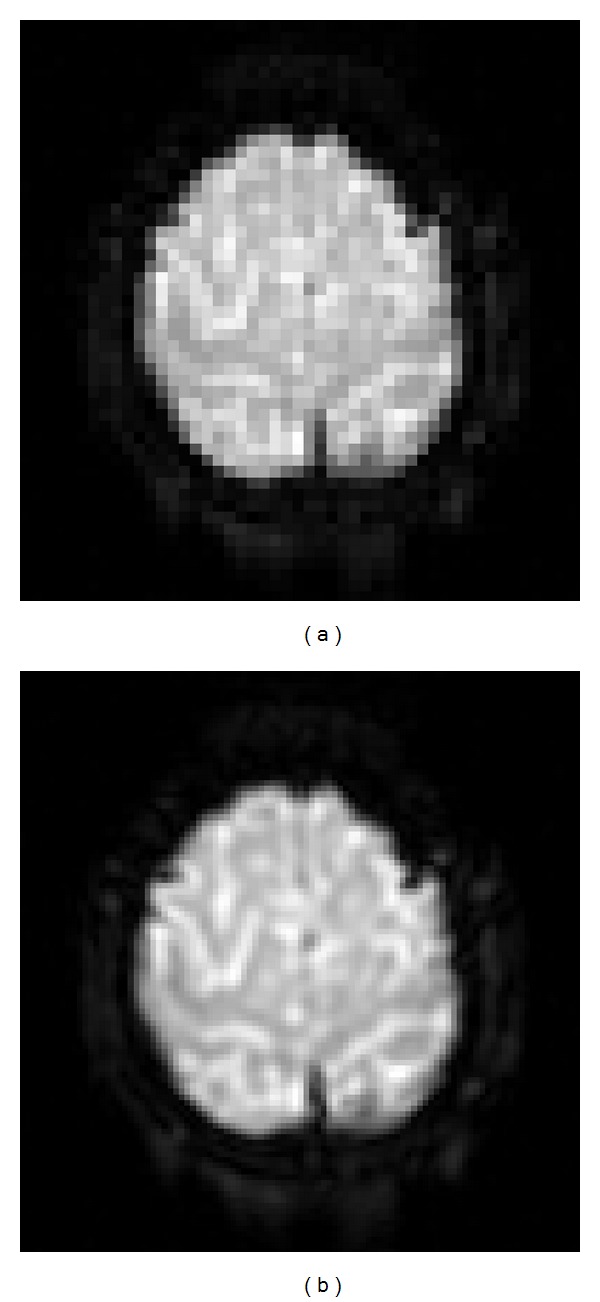
A comparison of the original (a) and VSI interpolated (b) echo-planar images shows that VSI improves delineation between the gray and white matter. In order to show these two images in the same size, each voxel in the original image was duplicated by a factor of 4 to form a 128 × 128 image in (a). The edge of the brain also appears much smoother in the interpolated image than in the original image.

VSI also improves the delineation of GM and white matter (WM) in the functional images, as shown in Figure 10. The thickness of GM in human brains is on the order of 2 mm, smaller than the typical voxel size used in fMRI. The shape of the GM regions is less continuous in the original EPI data due to PVE or the lack of sampling density in the image space. After VSI, the GM regions in the EPI data became clearer and more continuous. This improved delineation between GM and WM can lead to (1) more accurate coregistration between the EPI volumes at different time points during motion correction, (2) more accurate coregistration between the EPI data and the high-resolution anatomical data, and (3) improved warping of the EPI data onto standard brain template, such as the Montreal Neurological Institute (MNI) template.

Further increasing the matrix size with an interpolation factor of four (e.g., from 64 × 64 to 256 × 256) or higher may slightly reduce PVE and improve the detection of fMRI signal. However, the additional improvement is expected to be much less substantial than the improvement obtained with an interpolation factor of two (e.g., from 64 × 64 to 128 × 128) [13]. The penalty in computation time and the increased size of a dataset at a higher interpolation factors can easily outweigh the benefit of a more reduced PVE.

A direct comparison between the VSI activation maps and the ones obtained from high-resolution acquisition would be helpful for the validation of the proposed technique. Since high-resolution fMRI scans were not performed in the current study, such a comparison should be considered in future studies.

## 5. Conclusions

VSI improves the spatial accuracy of activation in fMRI through reducing PVE. Additionally, VSI can improve the sensitivity in detecting BOLD signal, especially when the size of activated regions is on the order of voxel size. VSI achieves these advantages of higher resolution fMRI without penalties in SNR, temporal resolution, and image artifacts.
